# Effectiveness and experiences of quality improvement interventions in older care: a mixed-methods systematic review

**DOI:** 10.3389/fpubh.2026.1813536

**Published:** 2026-04-30

**Authors:** Md. Shafiqur Rahman Jabin, Nussrat Bi, Ayesha Mirza, Marcus Chilaka, Emilia Vann Yaroson, Ray Samuriwo

**Affiliations:** 1 Department of Medicine and Optometry, Linnaeus University, Kalmar, Sweden; 2 Faculty of Health Studies, University of Bradford, Bradford, United Kingdom; 3 Faculty of Engineering and Digital Technologies, University of Bradford, Bradford, United Kingdom; 4 Management School, University of Sheffield, Sheffield, United Kingdom; 5 School of Health and Social Care, Edinburgh Napier University, Edinburgh, United Kingdom

**Keywords:** accessibility, active ageing, co-design, communication, health and well-being promotion, patient safety, person-centred care, risk reduction

## Abstract

**Background:**

The growing global population of older adults presents significant challenges for health and social care systems, requiring effective quality improvement (QI) interventions to enhance care delivery and outcomes. While various QI strategies have been implemented across care settings, evidence regarding their effectiveness and implementation experiences remains fragmented.

**Objective:**

This mixed-methods systematic review aims to examine both the effectiveness and experiences of QI interventions designed to improve care for older adults across diverse healthcare settings.

**Methods:**

A comprehensive search was conducted across multiple bibliographic databases and supplementary sources for studies published from 1990 onward. Studies were included if they reported an explicit QI intervention with implementation and evaluation components. Both quantitative and qualitative studies were included, and findings were synthesized using a convergent integrated approach. Data extraction and critical appraisal were conducted using established methodological frameworks.

**Results:**

A total of 23 studies were included, comprising nine qualitative, 12 quantitative, and two mixed-methods studies. QI interventions were categorized into key domains, including education and training, communication and collaboration, technology-based interventions, person-centered care, and health and well-being promotion. Quantitative findings demonstrated improvements in outcomes such as staff competence, patient safety, quality of life, and care coordination. Qualitative findings highlighted the importance of user engagement, contextual fit, communication, and organizational support in the successful implementation of QI interventions. Technology-based interventions showed potential benefits but were highly dependent on usability, training, and integration into care practices.

**Conclusion:**

QI interventions can improve outcomes and care experiences for older adults when implemented within supportive and context-sensitive care systems. No single intervention type is sufficient in isolation; rather, effective improvement requires a combination of strategies tailored to user needs and care contexts. Future research should focus on strengthening implementation processes and evaluating long-term sustainability across diverse settings.

**Systematic Review Registration:**

https://doi.org/10.2196/56346.

## Introduction

### Overview

Quality improvement (QI) interventions are not solitary endeavors but collaborative efforts involving various professionals and stakeholders. This collective approach aims to make positive changes in improving health and social care systems for older adults (1, 2). QI is a vital, continuous approach that designs, tests, and implements changes using real-time measurements. It significantly enhances the safety, effectiveness, and care experience for older adults (3). QI interventions are designed to illuminate and address the current challenges in caring for older individuals within healthcare and social care systems. These may include difficulties with staff working conditions for the organization as a whole [4]; the roles of older individuals in their own care, including support from relatives and families (4); and the roles of clinical and public health researchers, academics, engineers, and planning experts. These professionals play a crucial and valued role in designing, implementing, and evaluating QI interventions in older care, ensuring that these interventions are evidence-based, feasible, and sustainable (1, 5–7). For the purpose of this review, quality improvement (QI) interventions are defined as systematic, data-driven efforts to enhance healthcare processes, outcomes, or system performance through structured change and evaluation. These interventions typically involve iterative testing, measurement, and implementation of changes within real-world care settings (8, 9).

The global population of older adults is rapidly increasing due to improvements in life expectancy and public health (10). This demographic shift presents significant challenges for health and social care systems worldwide, including rising demand for long-term care, the management of multiple chronic conditions, and the need for integrated, person-centered services (11, 12). Across both high- and middle-income countries, there is growing recognition of the need to strengthen care delivery models to support aging populations effectively (13). For example, in Sweden, over 18% of the population is aged 65 or older, and this proportion is expected to continue rising in the coming decades (14). Reforms such as the ÄDEL reform have shifted care toward home-based services, improving accessibility and reducing reliance on institutional care (15, 16). However, challenges remain, particularly in the implementation and evaluation of new technologies and care models, including issues related to unequal access and unstructured implementation processes (17, 18), highlighting the ongoing need for effective quality improvement interventions in older care.

Several QI programs have shown promising results in improving the quality of health and social care for the aging population. For example, a QI program, such as Acute Care for Older Adults in the UK, not only improved the outcomes for older adults but also significantly reduced cost and length of stay, offering a promising avenue for potential cost savings (19). Other QI interventions, such as education sessions/toolkits, improved the impact of accurate and suitable medicines supply to the residents of residential aged-care facilities (20). A discharge planning intervention increased the feasibility and effectiveness of facilitating the transition of older adults from hospitals to their homes (21). An intervention comprising technical components such as assistive technologies (22) and social components like social interactions and connections (23) reduced preventable harm in care homes (24, 25). QI programs often involve collaboration between geriatricians and primary care physicians. This comprehensive approach not only improves the quality of care but also reduces hospitalization risk and total health and care costs among vulnerable older adults, providing a sense of reassurance about the effectiveness of these programs (26).

The review considered various dimensions of care quality, such as accessibility, safety, effectiveness, and patient-centeredness (11, 12, 27). Each dimension has been covered to provide comprehensive insight into health and care quality, underscoring the importance of a holistic approach (28). It is crucial to understand that the care provided should be adequate across all these dimensions; i.e., one or more missing aspects of the care do not complete the quality (29, 30). For instance, providing inappropriate or ineffective care should be unacceptable, even if it is safe and provided on time (28, 31, 32). This comprehensive understanding is urgent and necessary for improving health services.

Previous systematic reviews have examined quality improvement (QI) interventions within specific contexts or using single methodological approaches, such as quantitative or qualitative designs. For example, a mixed-methods systematic review in the radiology setting demonstrated improvements in workflow efficiency, communication, and patient safety (33, 34). These studies also highlight the importance of clearly defining QI interventions as structured efforts to improve care processes and outcomes through implementation and evaluation (8, 9). However, no comprehensive mixed-methods systematic review was identified that specifically examines both the effectiveness and experiences of QI interventions in older care settings. Therefore, this review aims to address this gap by integrating both quantitative and qualitative evidence.

This review specifically focused on the QI interventions in older care, including patient safety, appropriateness, and accessibility. These interventions could range from technological advancements to training programs, which could revolutionize older care through remote monitoring and telemedicine, and training, which is essential for equipping care providers with the necessary skills and knowledge (35) and training (36). However, the need for a comprehensive systematic review that examines the health and social care system for older adults from multiple lenses, including both quantitative and qualitative studies, is evident. Our review, with its broader perspectives, filled this gap and further facilitated the analysis of the issues and the devising of recommendations. A preliminary search has been conducted on Campbell Systematic Reviews, the Cochrane Database of Systematic Reviews, PROSPERO (International Prospective Register of Systematic Reviews), and JBI Evidence Synthesis.

### Aim and review questions

The primary purpose of this review is to compile and synthesize the best available evidence regarding the effectiveness of QI interventions in older care. This research has the potential to significantly impact the quality of care for older adults. It is a collective effort by healthcare professionals, researchers, policymakers, and stakeholders in older care, each of whom plays a crucial role. Understanding that QI initiatives ultimately target the beliefs and perspectives of clinicians and social workers, as well as the organization and its resources. The secondary objective of this review is to understand the experiences and perspectives of care providers, older individuals, their families, and relevant stakeholders involved in a QI initiative. By synthesizing existing literature and identifying knowledge gaps, particularly as articulated in the protocol by Jabin et al. in 2024, this study aims to assess the effectiveness of QI interventions in older care (37). Specifically, the review questions are:

- What QI interventions have been implemented to enhance the quality of care for older adults?
- How effective are interventions concerning policy and practice that target improvements in the quality of older care?
- What are the experiences and perspectives of care providers, older individuals and their families, and other relevant stakeholders, such as policymakers, healthcare administrators, and community leaders, regarding these interventions?

## Methods

The systematic review was conducted systematically, following the JBI methodology used as a guiding framework to inform the development of the inclusion criteria, allowing flexibility to capture the complexity and diversity of QI interventions, rather than as a rigid or prescriptive structure (38).

### Search strategy

The search strategy was designed to be comprehensive, including all relevant studies on interventions for older care. Databases were searched for both published and unpublished studies, following the standard three-step method for a systematic review (34, 37). The first step was an initial, limited search of relevant databases, followed by analysis of the words in the title and abstract, as well as the index terms used to describe the article. The search for published studies included a two-way search strategy. One approach was to search the journal and reference databases, including CINAHL, MEDLINE, PsycInfo, and Web of Science. Another approach was to search article-based (journal) databases, such as the ACM Digital Library, IEEE Xplore, and BMJ Journals. The search for unpublished studies (grey literature) included Mednar, Trove, OCLC WorldCat, and Dissertations and Theses. A second search was undertaken using all identified keywords (see Box 1) and index terms across all included databases. Additional search strategies, including citation searches for specific researchers or articles (e.g., gold-standard articles) and chain searches (reviewing the reference lists of systematically selected articles), were also employed to complement the search for published and unpublished papers. Studies, including reviews (systematic, scoping, and umbrella), and editorials were excluded. Any studies that did not raise ethical concerns were also excluded.

Box 1 **A list of keywords for the search strategy**

Participants: old people (patient), older people (patient), elderly people (patient), elderly people (patient), elderlies, ageing population, geriatricians, domiciliary workers (Using OR Boolean operator).

Context: elderly care, aged care, primary care, private hospital, public hospital, clinic, geriatric ward, old-age home, nursing homes, domiciliary care, home care (Using OR Boolean operator).

Interventions: Technology, training, education, staff arrangement, incident reporting, peer review, clinical audit, teamwork interventions, communication, safety checklists, and local governance. (Using OR Boolean operator).

Outcomes: Safety culture, decision-making, communication, teamwork, leadership, report turnaround time, and timeliness of care. (Using OR Boolean operator).

Types of Studies: Randomized controlled trials, cluster randomised controlled trials, quasi-experimental, controlled before and after trials, interrupted time series analysis, qualitative, grounded theory, ethnography, phenomenology, case study, narrative model, historical model (Using OR Boolean operator).

**Figure 1:**
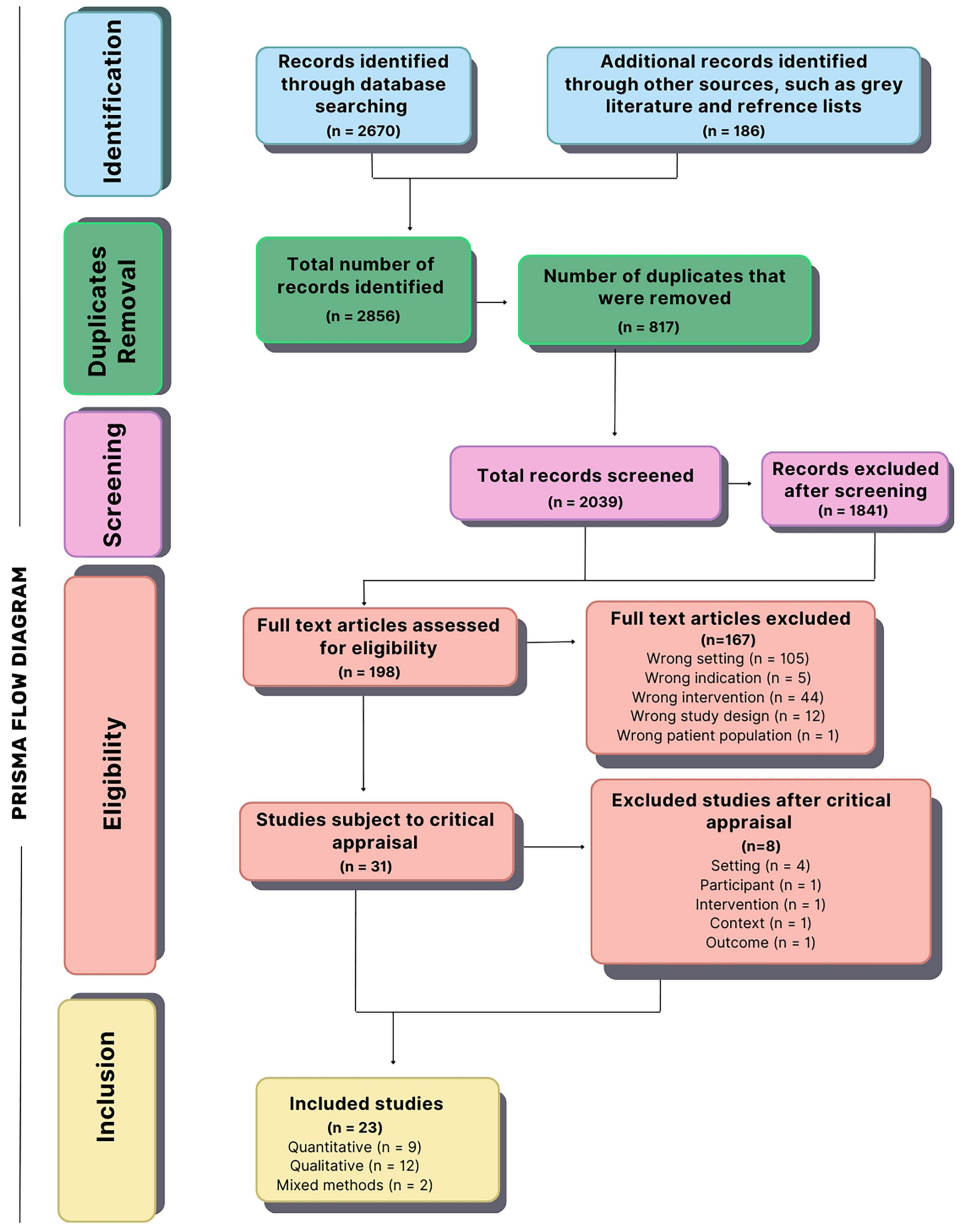
PRISMA flow diagram of included studies.

Only studies published in English were included due to resource constraints and to ensure consistency in data extraction and interpretation. The search was limited to studies published from 1990 onward, reflecting the emergence of modern patient safety and quality improvement research following the Harvard Medical Practice Study (39, 40). The results of the search strategy were visually depicted using the Preferred Reporting Items for Systematic Reviews (PRISMA) flow diagram (see Figure 1), providing a clear overview of the review process (41).

Search terms within each concept (Participants, Context, Interventions, Outcomes, and Types of study) were combined using the Boolean operator ‘OR’, while the different concepts were combined using ‘AND’ to generate comprehensive search strings across databases.

### Inclusion criteria

The inclusion criteria of this review were designed to be inclusive, ensuring that all relevant studies were considered. We followed the PICO (quantitative studies) and/or PICo (qualitative studies) mnemonics, which stand for Population, Phenomenon of Interest, and Context. These mnemonics were used as a guide (not policy); therefore, the inclusion criteria of this systematic review included a detailed description of types of participants/ population, types of interventions, phenomena of interest, context, outcomes, as well as types of studies, search strategies, assessment of methodological quality, and synthesis of results. Our inclusive approach to the criteria ensured that we considered a wide range of studies, providing a comprehensive view of the topic.

#### Types of participants/population

This review included studies of older individuals (aged 65 years or above) undergoing older care irrespective of gender and diversity, including age, ethnicity, socio-economic status, and disability and any health and care professionals (care providers) and stakeholders involved in the care delivery for older individuals, such as nurse practitioners, registered nurses, enrolled nurses, specialist nurses, and geriatricians.

#### Types of interventions

The included studies focused on implementing a QI intervention, i.e., a systematic, continuous approach that designs, tests, and implements changes using real-time measurements to improve the safety, effectiveness, and experience of care for older adults. Given the complexities of the health and social care system, this review included a range of QI interventions.

To be eligible for inclusion, studies were required to report an explicit QI intervention, defined as a structured initiative aimed at improving care processes or outcomes, supported by an implementation component and an evaluative element (e.g., pre–post assessment, outcome measurement, or process evaluation).

Studies that solely explored perceptions, experiences, or attitudes toward care or technology without the implementation or evaluation of a QI intervention were excluded unless these were directly linked to an ongoing or completed QI initiative. This ensured that all qualitative evidence included in the review was directly relevant to understanding the implementation, experience, or outcomes of QI interventions.

#### Phenomena of interest

The phenomena of interest were the effects on outcomes and workflow processes, as well as the experiences and perspectives of care providers and older individuals undergoing or being exposed to the QI interventions. These experiences and perspectives of care providers and older individuals regarding the implementation and outcomes of QI interventions, workplace contexts and cultures (including factors such as conflict and its management, teamwork behaviors, and care providers’ attitudes), and the management of adverse events and near misses.

#### Context

The systematic review considered studies conducted in older care settings, including geriatric wards in primary healthcare, hospitals or clinics, old-age homes, nursing homes, and home care facilities for older people.

#### Outcomes

The outcomes included validated measures of safety culture, decision-making, communication, teamwork, leadership, report turnaround time, and timeliness of care. Further outcomes were patient satisfaction and patients’ perceptions of the quality of older care (42).

### Types of studies

Quantitative and qualitative studies were included to report empirical evidence and the human experience. Quantitative studies include Randomized Controlled Trials (RCTs) or Cluster Randomized Controlled Trials (CRCTs); non-randomized controlled trials (NRCTs); quasi-experimental, controlled before-and-after (CBA) trials; and interrupted time series studies. Qualitative studies included interpretive work focusing on, but not limited to, designs such as content analysis, phenomenology, grounded theory, ethnography, case study, narrative model, and historical model. Mixed methods and descriptive studies will also be eligible for inclusion.

The selection of study designs was guided by the Joanna Briggs Institute (JBI) methodology for mixed-methods systematic reviews, which supports the inclusion of a broad range of quantitative and qualitative evidence to address complex healthcare interventions (43).

### Assessment of methodological quality

Two independent reviewers assessed the methodological validity before inclusion and selected quantitative and qualitative papers for retrieval (NB as the primary reviewer of all studies, and MSRJ and AM as the secondary reviewers), thereby reducing the risk of methodological issues, such as subjective bias. This was done using the standardized critical appraisal criteria from the critical appraisal instruments by the Joanna Briggs Institute (JBI) (44). Any discrepancies between reviewers’ assessments were resolved by consensus through discussion.

A critical appraisal of the selected papers will be conducted after screening. The GRADE (Grading of Recommendations Assessment, Development and Evaluation) and GRADE-CERQual (Confidence in the Evidence from Reviews of Qualitative Research) frameworks were used to rate the quality of scientific evidence and the confidence in findings from systematic reviews of both quantitative and qualitative studies, respectively. For example, three quality grades were used for each study, based on the score achieved in the critical appraisals: low quality (less than 33% or equal to 33%), medium quality (34–66%), and high quality (more than 66%).

### Data extraction

Quantitative and qualitative data were extracted from papers included in the review with the utmost care and precision. In this case, we used the Covidence systematic Review tool (due to institutional access limitations to the JBI SUMARI tool at the time of the review) for data extraction to ensure consistency and thoroughness (45). The data extracted for quantitative and qualitative studies included details on interventions, populations, study methods, and outcomes that were relevant to the review question and objectives. This meticulous data extraction process, conducted with a keen eye for detail, ensured the accuracy and reliability of our findings. Our commitment to precision in data extraction instilled confidence in the robustness of our review.

## Results

### Search strategies and screening

The results of the search strategy, appraisal, and inclusion of studies are summarized in Figure 1, presented as a PRISMA flow diagram. A total of 2,856 records were identified, including those from bibliographic databases (*n* = 2,670) and grey literature and other sources, such as reference lists (*n* = 186). After removing 817 duplicates, 2,039 records were screened. The titles and abstracts of these studies were independently screened by multiple reviewers (NB as the primary reviewer, with MSRJ and AM as secondary reviewers). Of the 2,039 studies, 1,841 were excluded as they were not relevant to the review question. A full-text review of the remaining 198 studies resulted in 31 studies progressing to critical appraisal, while 167 were excluded for not meeting one or more eligibility criteria (e.g., incorrect setting, intervention, study design, or population). Of the 31 studies appraised, 23 were ultimately included in the review following the screening and selection process, comprising nine qualitative, 12 quantitative, and two mixed-methods studies (Figure 1).

For example, some studies initially appeared relevant because they focused on older adults and care technologies; however, they were excluded because they did not involve an explicit quality improvement (QI) intervention or did not address the review’s research questions related to intervention effectiveness or implementation experiences.

### Critical appraisal results for included studies

Joanna Briggs Institute critical appraisal checklists for RCTs, quasi-experimental studies, and qualitative studies are presented in Appendix Table 1. Critical appraisal results for the five RCTs (46–50); five quasi-experimental studies (21, 51–54); and eight qualitative studies (55–62) are presented in Appendix Table 1.

All RCTs were of medium quality except for (50), which was rated as low quality. Questions C9, C10, C11, and C12 had the highest positive responses regarding outcome measures and their analysis (Appendix Table 1A). Of the six quantitative non-RCTs, four were found to be of high quality (21, 51, 52, 54) and one of low quality (53). Questions C1, C3, and C7 received the highest positive responses regarding cause-effect variables, participant involvement, and outcomes (Appendix Table 1B). Of the eight qualitative studies, seven were found to be of high quality, including: (55, 57–62), and one of medium quality (56). Questions C4, C5, and C10 received 100% positive responses regarding research methodology and the analysis of research data (Appendix Table 1C).

### Summary of the characteristics of the included studies

#### Location

Studies were conducted in 12 countries: The United States of America, the United Kingdom, Canada, Denmark, Finland, the Netherlands, Iran, Japan, China, Spain, Italy, and Indonesia. The United States contributed the most studies (*n* = 5) (21, 47, 48, 62, 63), followed by the United Kingdom (*n* = 3) (51, 56, 58), Iran (*n* = 3) (46, 53, 64), and Canada (*n* = 1) (49). There are other countries in the study, including Denmark (59), the Netherlands (50), Finland (52), China (61), Japan, (65), Spain (57), Italy, (66), and Indonesia, (67), each of which contributed one or two studies. This international distribution highlights the engagement of both high- and middle-income countries in QI for home- and community-based care for older adults (see Tables 1, 2).

**Table 1:** Summary of the included studies.

**No.**	**Author(s)/year**	**Aims and/or objectives**	**QL/QT/mixed (quality)**	**Study population and context**	**Type of QI intervention**	**Main findings/conclusion**	**Further suggestions**
1	Rooijackers et al (2022) (50) Netherlands	-To change staff behavior from “Doing for” to “Doing with” older adults, i.e.,supporting client activation	QT High	313 participants, ten Dutch homecare nursing teams, comprising 135 nursing team members and 178 domestic workers	Training the staff in reablement to shift from “doing for” to “doingwith” older adults)- Rooijackers et al. (50)	-The study does not demonstrate beneficial effects for the SAaH reablement training program on home care staff's self-efficacy and outcome expectations regarding client activation -Nonetheless, reablement seems to be “the right thing to do”, especially in light of the challenges of the aging population	-Refining the training and supporting staff through mentorship could improve outcomes -Long-term research and collaboration may also help
2	Yu et al (2023) (61) China	-To explore the reasons for the long-term adoption or abandonment of technology behaviors by elders in the home care environment.	QL Low	26 elders	Technology: adoption of smart technology by older adults	-The study explores the reasons for the long-term adoption and abandonment of smart technology by the elderly in the home care environment -The research shows that whether the elderly adopt smart technology depends on the user’s experience.	-Personalized training and simple designs can improve adoption -Ongoing support and caregiver involvement ensure long-term use.
3	Petersen et al (2019) (59) Denmark	-To explore how the registered nurses at the hospital and in home care talk about and experience cross‐sectoral collaboration related to transitional care of frail older patients	QL Medium	79 nurses from three medical and surgical hospital wards	Communication (between nurses in different healthcare sectors)	The study identified one main theme, “It is two worlds”, and three subthemes: “Different objectives and approaches to nursing in the two worlds," “Assumptions and preconceptions in the two worlds”, and “Interaction and collaboration between nurses in the two worlds"	-Clarifying roles and addressing preconceptions can improve collaboration -Regular communication and joint training can strengthen teamwork.
4	Olatunji et al (2024) (58) United Kingdom	-To develop and customize assistive robots that effectively support older adults in their daily activities by actively involving them and their care partners in an immersive participatory design process	QL Low	Two older adults, an occupational therapist and robotics engineer, are embedding themselves in the participants’ homes	Assistive robotics co-designed with users	-The study highlights the significance of co-designing assistive technologies with end-users to achieve successful and meaningful outcomes.	-Involve diverse users, use continuous feedback during development, and offer ongoing support.
5	Davodi et al (2023) (46) Iran	-To determine the effectiveness of an intervention program and promote active aging in the elderly, referred to the Mashhad Health Centers	QT Low	60 elderly individuals without disabling diseases and cognitive impairment	Assistive technology for active aging	-The findings show a practical approach to promote active aging as an intervention at the level of health centers.	-Incorporate social activities, tailor wellness programs, and involve family and caregivers in active aging
6	Hoben et al (2020) (47) Canada	-To compare the effects of different feedback interventions on improving formal staff communication among care aides and nurses in nursing homes, explicitly assessing two higher-intensity feedback interventions against a simple feedback approach	QT High	4,641 care aides and 1,693 nurses, totaling 6,334 participants across 67 nursing homes	Communication (among care aides and nurses)	-The study's findings indicate that higher-intensity feedback interventions (basic and enhanced) significantly improved care aide involvement in formal communications about resident care, compared to the simple feedback control group, with no significant difference in effectiveness between the two higher-intensity interventions -The conclusion emphasizes that theory-based feedback can enhance communication among care teams in nursing homes, which is crucial for improving residents' quality of care and safety	-Standardize feedback to improve consistency, and personalize feedback based on individual care aide roles to increase effectiveness -Offer additional communication training to ensure better application of feedback in daily care.
7	Keller et al (2017) (62) United States	-To evaluate the barriers and facilitators to the appropriate use of home medical devices by older adults during the transition from hospital to skilled home health care (SHHC)	QL Medium	63 participants: 24 older adults and their informal caregivers 39 skilled home health care providers (SHHCPs) and administrators	Medical devices for hospital-to-home transition	The study concludes that integrating care, providing adequate training, optimizing home environments, ensuring clear communication, and conducting regular assessments are essential for the safe and effective use of home medical devices by older adults	-Involve family in training and assessment to support older people at home, use digital tools or apps to assist with device management and reminders. -Provide ongoing follow-up services to monitor device use and address issues.
8	Pigini et al (2012) (66) Italy	-To generate user requirements and realistic usage scenarios, maximizing alignment with users’ needs, perceptions, feelings, and rights.	Mixed (QL+QT) High	Focus Groups: 22 elderly persons participated, alongside 17 relatives and 20 health professionals Individual Interviews: 64 elderly persons, 19 family members, and 46 caregivers participated	Robotic assistance for elderly care	The study highlights that elderly participants face significant challenges in daily living, especially with mobility and memory, while they were generally open to using a remote-controlled robot, caregivers expressed privacy concerns -Health professionals perceive potential benefits for communication between elderly users and families -Overall, the findings highlight the need for tailored robotic solutions that address user needs and concerns.	-Design robots with privacy features to address caregiver concerns, customise robot functions to meet individual needs, like memory prompts -Involve caregivers in the design process to ensure the technology supports both users and caregivers effectively
9	Markle-Reid et al (2013) (49) Canada	-To evaluate the effectiveness of different multi-component nurse-led health promotion and disease prevention (HPDP) interventions compared to usual home care among frail older adults	QT High	498 participants, these were community-living frail older adults (aged 65 years and older)	Education (Informing older adults and caregivers on health management)	-The study finds that nurse-led health promotion interventions significantly improve health-related quality of life (HRQOL) for frail older adults compared to usual home care These interventions are well-accepted, feasible to implement, and effective when they include multiple home visits and inter-professional collaboration -The findings emphasize the need for further research and investment in such interventions to enhance care for this population.	-Expand collaboration with more professionals, incorporate technology, like telehealth, for remote care monitoring and continuous care -Tailor interventions to individual needs and preferences to enhance engagement and outcomes
10	Pakkonen et al (2024) (54) Finland	-To evaluate the effectiveness of a continuing education (CE) intervention named ‘Person First Please’ (PFP) for improving nurses' PPC competence and its connection to PPC climate	QT Medium	200 professional nurses participated, 77 in the intervention group and 123 in the control group	Education (Person First Please (PFP) continuing education program)	-The study concludes that person-centered care (PCC) competence significantly increases in the intervention group and remains after 6 weeks of follow-up. -PCC climate increases in the intervention group in total score and all sub-scales across residents with their next of kin -The control group does not show any significant change. Comparisons of PCC competence and PCC climate between intervention and control groups confirm that changes between groups are statistically significant in the intervention group	-Maintain follow-up training, expand the intervention to include more healthcare professionals for a broader impact, and incorporate feedback from residents and their families to refine and sustain effective PCC practices.
11	Macías-Colorado et al (2021) (57) Spain	-To describe the factors influencing the process followed by community nurse case managers and provide communication on safe caregiving to family members caring for dependent elderly individuals	QL Low	Seven community nurse case managers (CNCMs) from seven urban healthcare facilities	Communication (about safe caregiving between CNCMs and FCs)	-The study finds that home care by family members involves significant risks, particularly due to challenges in communication between community nurse case managers (CNCMs) and family caregivers (FCs) -Effective interventions must consider nurses' professional skills, caregiving dynamics, and the home environment -Communication difficulties are exacerbated when multiple caregivers are involved, leading to increased risks. To improve safety, it is crucial to adapt information for clarity, implement verification tools, and utilize new technologies -Additionally, exploring CNCMs' and FCs' perceptions of safety can help develop tailored home care strategies	-Provide communication training to improve clarity and reduce miscommunication -Use digital tools for coordination. Develop standardized care protocols for multi-caregiver situations to enhance consistency and safety.
12	Light et al (2016) (48) United States	-To determine the benefit of a weekly telephone contact on balance control for community-dwelling frail older adults participating in home-exercise programs. -To improve adherence to the exercise program and, consequently, enhance outcomes on the Berg Balance Test	QT Low	75 participants who were community-dwelling, frail older adults	Training (adherence to the exercise program)	-The key findings indicate that the Telephone Call group improved balance control significantly more (6.3 points) than the No Telephone Call group (3.9 points) on the Berg Balance Scale -After four weeks, the Telephone Call group continues to progress, while the No Telephone Call group plateaus -The Telephone Call group has a lower dropout rate, indicating better adherence. Overall, weekly phone contact enhances the effectiveness of home-exercise programs for frail older adults at risk of falling	-Increase call frequency -Tailor phone calls to address challenges and provide personalized support -Combine calls with additional remote support, such as video check-ins, for enhanced effectiveness
13	der Cingel et al (2021) (55) Netherlands	-To gain insight into how home care nurses assess eHealth interventions during their assessment of care -To understand nurses' practices regarding eHealth to support them in effectively integrating these interventions into homecare settings.	QL Medium	43 participants (home care nurses with varying levels of experience): 12 in the "think-aloud" interviews, 6 in the orientation phase, and 12 in a focus group for member checking	Training and Technology (in assessing and implementing eHealth solutions)	-The study's main findings indicate that nurses require a trusting relationship with clients to suggest eHealth interventions and that home visits are essential for assessing these technologies. -Many nurses express skepticism about the suitability of eHealth for frail older adults -The study concludes that for nurses to assess and utilize eHealth effectively, it must be seen as a fit for clients' needs in a person-centered way -Additionally, the research highlights the necessity for training and tools to keep nurses informed about relevant eHealth innovations	-Train nurses on eHealth to build trust and confidence in recommending it. Ensure eHealth tools are tailored to individual needs for better integration into person-centered care -Offer continuous support and resources to inform nurses about the latest eHealth innovations
14	Dedhia et al (2009) (21) United States	-To study the feasibility and effectiveness of a discharge planning intervention	QT High	422 participants: 237 patients were followed during the pre-intervention period, and 185 patients were exposed to the intervention	Planning Intervention	-The study concludes that hospitalized elderly patients can significantly improve their healthcare outcomes when their specific needs are considered through a structured discharge planning intervention -This emphasizes the importance of tailored discharge strategies for enhancing patient transitions from hospital to home	-Involve family members in the discharge process to ensure they are prepared to assist at home -Provide follow-up support and connect patients with community resources to aid their transition and post-hospital care
15	Vilstrup et al (2017) (60) Denmark	-To explore the perspectives of older care recipients regarding the use of iPads by home care nurses. -To understand how patients perceive the integration of this technology in their care and its impact on the nurse-patient relationship	QL Low	Seven participants who were receiving home care, aged 62 to 90 years	Technology (use of iPads by home care nurses)	-The study concludes that patients have a more positive or indifferent view of technology like iPads in home care, contrasting with the nursing perspective that often sees technology as "cold" -The findings suggest that technology can enhance care and communication without compromising the nurse-patient relationship -Future research should further explore both patient and nurse perspectives on technology use in healthcare	-Provide training for nurses on integrating technology without compromising the personal care aspect -Encourage collaborative use of technology between nurses and patients to enhance communication -Explore how technology impacts the aspects of patient care.
16	Kajander-Unkuri et al (2022) (52) Finland	-To investigate the effectiveness of combined web-based and simulation-based continuing education on home-care professionals' competence in evaluating the acute care needs of older people	QT Medium	254 home-care professionals participated in the combined web-based and simulation-based continuing education, of which 171 participants completed the online survey before the education, while 83 participants completed the survey after the education	Education and Training (Providing ongoing education to healthcare professionals to improve their skills and knowledge)	-The study finds that the combined web-based and simulation-based continuing education significantly improved home-care professionals' competence in evaluating older people's acute care needs -The mean competence score increased from 3.22 before the education to 3.92 after the education -Improvements are observed in overall competence and across all eight subscales measured, with the most significant gains in health assessment and consultation -This indicates that such educational interventions are effective in enhancing the skills required for a timely and accurate evaluation of older adults' health conditions	-Expand course content to include emerging healthcare trends and technologies. Offer refresher courses to maintain and update professional skills -Encourage peer learning through online forums or group simulations to enhance collaboration and knowledge sharing
17	Hall et al (2011) (56) United Kingdom	-To explore the perceived benefits and barriers to implementing the Gold Standards Framework for Care Homes (GSFCH), a quality improvement program in palliative care -To gather views from care home staff, residents, and their families to inform the development of better palliative care interventions in care homes for older people	QL Medium	44 participants: nine care home managers, eight nurses, nine care assistants, 11 residents, and family members of residents	Education and Training (for care staff)	The study finds that while seven of nine care homes are positively progressing with the Gold Standards Framework for Care Homes (GSFCH), they face significant barriers to full implementation, including communication issues with primary care, staff workload, and resource limitations -The findings highlight the need for strategies to overcome barriers and further research on the program's impact on residents and families	-Improve communication between care homes and primary care providers to enhance coordination. Hire more staff or offer additional training to address workload challenges.
18	Tanaka et al (2024) (65) Japan	-To explore the perspectives of Japanese elders, their caretakers, and healthcare providers on using wearable technology to monitor health at home	QL Low	21 participants, including Japanese elders, their caretakers, and healthcare providers	Wearable technologies	The study finds that elderly participants are interested in wearable devices but face barriers like cost and complexity -It identifies four main themes: health issues, use of technology, and the advantages and disadvantages of wearable devices -The study concludes that these devices can benefit elderly individuals and require more research	-Simplify wearable designs and make them affordable -Ongoing training and technical support for both users and healthcare providers -Integrating wearables with existing healthcare systems and offering customizable features
19	Shagerdi et al (2022) (64) Iran	-To explore opportunities for using health information technology to improve elderly care in emergency departments -To assess current care processes, identify necessary data, and evaluate existing technologies -To focus on how technology could address challenges like workload and improve collaboration in elderly care	QL Medium	33 healthcare professionals, including geriatricians, geriatric nurses, emergency medicine specialists, and emergency department nurses, conducted in emergency departments focusing on elderly patient care.	Technology (Implementing health information technology)	-The study finds no specific workflow for elderly care in EDs and suggests that tailored health IT solutions could improve care	-Create health information technology systems for elderly care in emergency departments and train staff on their use -More research is needed to assess the impact on patient outcomes and staff efficiency
20	Damery et al (2021) (51) United Kingdom	-To evaluate the impact of the "Safer Provision and Caring Excellence" (SPACE) QI program on care processes, safety climate, and avoidable harms in care homes -To assess staff experiences and changes in working practices	Mixed (QL+QT) High	29 care homes: data were collected from 49 staff members across four case study homes (the total number of staff in all 29 care homes was not explicitly stated in the study)	Skills training and intensive support (Safer Provision and Caring Excellence (SPACE) program)	-The SPACE program improved safety climate and reduced avoidable harms like falls and pressure ulcers -It also decreases emergency hospital attendances, though admissions increased -Upskilling staff enhances teamwork and safety practices, but high turnover may affect long-term sustainability.	-Address staff turnover and provide ongoing training to new staff -Reinforcing safety practices is essential for sustaining improvements -Expand the program to other care homes to offer further insights.
21	Read et al (2021) (63) Canada	To explore using web-based videoconferencing (WBVC) for in-home palliative care consultations with elderly rural patients -To assess the feasibility, acceptability, and effectiveness of WBVC for providing care to these patients	QL Medium	27 participants in total: 10patients, 10 familymembers, and sevenhome care nurses,explored using web-basedvideoconferencing forpalliative careconsultations	Web-basedvideoconferencing	The study finds that web-based videoconferencing (WBVC)improves access to palliative care and is acceptable to patients,despite some technical issues -It saves time and travel, but raises concerns about data security -WBVC is a feasible alternative to in-person visits, but should complement them, not replace them.	-Improve provider training to reduce technical issues -Enhance technology to address quality and security concerns -Expand access to more rural areas to increase care reach.
22	Kousha et al (2024) (53) Iran	-To assess the effectiveness of an informal home care support program (HoSIP) in reducing loneliness and improving the quality of life among lonely older adults -To assess changes in health, social support, and self-care and the feasibility of implementing the program was examined	QT Low	36 participants (6 men and 30 women) were community-dwelling older adults aged 60 and above, who took part in the study in community settings.	Education and Training (Informal home care support intervention program)	The intervention reduces loneliness and improves quality of life through peer support and educational activities, and is a feasible and cost-effective way to combat loneliness in older adults	-Expand community involvement and digital literacy training to improve program reach -Increase follow-up support and add more activities to enhance effectiveness -Partnerships with local organizations can help sustain the program in the long term
23	Cahyanto et al (2023) (67) Indonesia	To evaluate the Spirit program for older adults in nursing homes, focusing on its reach, effectiveness, adoption, implementation, and maintenance using the RE-AIM framework -To assess participant engagement, perceived benefits, and the program’s sustainability	QT Low	Seven older adults and two caregivers, totalling nine participants, from a nursing home in Surakarta, Indonesia	Education and Training (physical exercises and mental health activities)	-The intervention improves participants' happiness, stamina, and sleep quality -Despite some challenges with balance exercises, the program is well-received with no injuries -It effectively enhances quality of life and can be sustained with accessible tools and consistency.	-Consider incorporating regular feedback to adapt the program and train more staff for better sustainability -Expand the program to include social interaction components, such as group activities or virtual meetings, which can further reduce isolation and improve overall well-being

CE, Continuing Education; CNCM, Community Nurse Case Manager; ED, Emergency Department; eHealth, Electronic Health; FC, Family Caregiver; GSFCH, Gold Standards Framework for Care Homes; HPDP, Health Promotion and Disease Prevention; HoSIP, Home Support Intervention Programme; HRQOL, Health-Related Quality of Life; IT, Information Technology; PCC, Person-Centred Care; PFP, Person First Please; PPC, Person-Patient Care; QI, Quality Improvement; QL, Qualitative; QT, Quantitative; RE-AIM, Reach, Effectiveness, Adoption, Implementation, and Maintenance; SAaH, Stay Active at Home; SHHC, Skilled Home Health Care; SHHCP, Skilled Home Health Care Provider; SPACE, Safer Provision and Caring Excellence; WBVC, Web-Based Videoconferencing.

**Table 2:** Key characteristics (extended) of included studies.

**No.**	**Author(s)/** **year/country**	**Area of application/purpose of QI**	**Main findings/conclusion**	**Further suggestions**
1	Rooijackers et al (2022) (50) Netherlands	Enhancing caregiver-client collaboration	-The study does not demonstrate beneficial effects for the SAaH reablement training program on home care staff's self-efficacy and outcome expectations regarding client activation -Nonetheless, reablement seems to be “the right thing to do”, especially in light of the challenges of the aging population	-Refining the training and supporting staff through mentorship could improve outcomes -Long-term research and collaboration may also help
2	Yu et al (2023) (61) China	Understanding technology adoption and abandonment by elders	-The study explores the reasons for the long-term adoption and abandonment of smart technology by the elderly in the home care environment -The research shows that whether the elderly adopt smart technology depends on the user’s experience.	-Personalized training and simple designs can improve adoption -Ongoing support and caregiver involvement ensure long-term use.
3	Petersen et al (2019) (59) Denmark	Examining cross-sector collaboration experiences	The study identified one central theme, “It is two worlds”, and three subthemes: “Different objectives and approaches to nursing in the two worlds," “Assumptions and preconceptions in the two worlds”, and “Interaction and collaboration between nurses in the two worlds"	-Clarifying roles and addressing preconceptions can improve collaboration -Regular communication and joint training can strengthen teamwork.
4	Olatunji et al (2024) (58) United Kingdom	Customizing assistive robots collaboratively	-The study highlights the significance of co-designing assistive technologies with end-users to achieve successful and meaningful outcomes	-Involve diverse users, use continuous feedback during development, and offer ongoing support
5	Davodi et al (2023) (46) Iran	Promoting active ageing	-The findings show a practical approach to Promote active aging as an intervention at the level of health centers	-Incorporate social activities, tailor wellness programs, and involve family and caregivers in active aging
6	Hoben et al (2020) (47) Canada	Improving the effectiveness of staff communication	-The study's findings indicate that higher-intensity feedback interventions (basic and enhanced) significantly improved care aide involvement in formal communications about resident care, compared to the simple feedback control group, with no significant difference in effectiveness between the two higher-intensity interventions -The conclusion emphasizes that theory-based feedback can enhance communication among care teams in nursing homes, which is crucial for improving residents' quality of care and safety	-Standardize feedback to improve consistency, and personalize input based on individual care aide roles to increase effectiveness -Offer additional communication training to ensure better application of feedback in daily care.
7	Keller et al (2017) (62) United States	Enhancing the safety and effectiveness of home medical devices	The study concludes that integrating care, providing adequate training, optimizing home environments, ensuring clear communication, and conducting regular assessments are essential for the safe and effective use of home medical devices by older adults	-Involve family in training and assessment to support older people at home, use digital tools or apps to assist with device management and reminders. -Provide ongoing follow-up services to monitor device use and address issues.
8	Pigini et al (2012) (66) Italy	Supporting and assisting	The study highlights that elderly participants face significant challenges in daily living, especially with mobility and memory, while they were generally open to using a remote-controlled robot, caregivers expressed privacy concerns -Health professionals perceive potential benefits for communication between elderly users and families -Overall, the findings highlight the need for tailored robotic solutions that address user needs and concerns.	-Design robots with privacy features to address caregiver concerns, customize robot functions to meet individual needs, like memory prompts -Involve caregivers in the design process to ensure the technology supports both users and caregivers effectively
9	Markle-Reid et al (2013) (49) Canada	Home care for frail older adults	-The study finds that nurse-led health promotion interventions significantly improve health-related quality of life (HRQOL) for frail older adults compared to usual home care These interventions are well-accepted, feasible to implement, and effective when theyinclude multiple home visits and inter-professional collaboration -The findings emphasize the need for further research and investment in such interventions to enhance care for this population.	-Expand collaboration with more professionals, incorporate technology, like telehealth, for remote care monitoring and continuous care -Tailor interventions to individual needs and preferences to enhance engagement and outcomes
10	Pakkonen et al (2004) (54) Finland	Supporting and assisting	-The study concludes that person-centered care (PCC) competence significantly increases in the intervention group and remains after 6 weeks of follow-up. -PCC climate increases in the intervention group in total score and all sub-scales across residents with their next of kin -The control group does not show any significant change. Comparisons of PCC competence and PCC climate between intervention and control groups confirm that changes between groups are statistically significant in the intervention group	-Maintain follow-up training, expand the intervention to include more healthcare professionals for a broader impact, and incorporate feedback from residents and their families to refine and sustain effective PCC practices.
11	Macías-Colorado et al (2021) (57) Spain	Safe caregiving	-The study finds that home care by family members involves significant risks, particularly due to challenges in communication between community nurse case managers (CNCMs) and family caregivers (FCs) -Effective interventions must consider nurses' professional skills, caregiving dynamics, and the home environment -Communication difficulties are exacerbated when multiple caregivers are involved, leading to increased risks. To improve safety, it is crucial to adapt information for clarity, implement verification tools, and utilize new technologies -Additionally, exploring CNCMs' and FCs' perceptions of safety can help develop tailored home care strategies	-Provide communication training to improve clarity and reduce miscommunication -Use digital tools for coordination. Develop standardized care protocols for multi-caregiver situations to enhance consistency and safety.
12	Light et al (2016) (48) United States	Education and Training	-The key findings indicate that the Telephone Call group improved balance control significantly more (6.3 points) than the No Telephone Call group (3.9 points) on the Berg Balance Scale -After four weeks, the Telephone Call group continues to progress, while the No Telephone Call group plateaus -The Telephone Call group has a lower dropout rate, indicating better adherence. Overall, weekly phone contact enhances the effectiveness of home-exercise programs for frail older adults at risk of falling	-Increase call frequency -Tailor phone calls to address challenges and provide personalized support -Combine calls with additional remote support, such as video check-ins, for enhanced effectiveness
13	der Cingel et al (2021) (55) Netherlands	Person-centred care	-The study's main findings indicate that nurses require a trusting relationship with clients to suggest eHealth interventions and that home visits are essential for assessing these technologies. -Many nurses express skepticism about the suitability of eHealth for frail older adults -The study concludes that for nurses to assess and utilize eHealth effectively, it must be seen as a fit for clients' needs in a person-centered way -Additionally, the research highlights the necessity for training and tools to keep nurses informed about relevant eHealth innovations	-Train nurses on eHealth to build trust and confidence in recommending it. Ensure eHealth tools are tailored to individual needs for better integration into person-centered care -Offer continuous support and resources to inform nurses about the latest eHealth innovations
14	Dedhia et al (2009) (21) United States	Transitional care	-The study concludes that hospitalized elderly patients can significantly improve their healthcare outcomes when their specific needs are considered through a structured discharge planning intervention -This emphasizes the importance of tailored discharge strategies for enhancing patient transitions from hospital to home	-Involve family members in the discharge process to ensure they are prepared to assist at home -Provide follow-up support and connect patients with community resources to aid their transition and post-hospital care
15	Vilstrup et al (2017) (60) Denmark	Patient Engagement (Using iPads to involve patients in their care)	-The study concludes that patients have a more positive or indifferent view of technology like iPads in home care, contrasting with the nursing perspective that often sees technology as "cold" -The findings suggest that technology can enhance care and communication without compromising the nurse-patient relationship -Future research should further explore both patient and nurse perspectives on technology use in healthcare	-Provide training for nurses on integrating technology without compromising the personal care aspect -Encourage collaborative use of technology between nurses and patients to enhance communication -Explore how technology impacts the aspects of patient care.
16	Kajander-Unkuri et al (2022) (52) Finland	Competence enhancement	-The study finds that the combined web-based and simulation-based continuing education significantly improved home-care professionals' competence in evaluating older people's acute care needs -The mean competence score increased from 3.22 before the education to 3.92 after the education -Improvements are observed in overall competence and across all eight subscales measured, with the most significant gains in health assessment and consultation -This indicates that such educational interventions are effective in enhancing the skills required for a timely and accurate evaluation of older adults' health conditions	-Expand course content to include emerging healthcare trends and technologies. Offer refresher courses to maintain and update professional skills -Encourage peer learning through online forums or group simulations to enhance collaboration and knowledge sharing
17	Hall et al (2011) (56) United Kingdom	End-of-life care	The study finds that while seven of nine care homes are positively progressing with the Gold Standards Framework for Care Homes (GSFCH), they face significant barriers to full implementation, including communication issues with primary care, staff workload, and resource limitations -The findings highlight the need for strategies to overcome barriers and further research on the program's impact on residents and families	-Improve communication between care homes and primary care providers to enhance coordination. Hire more staff or offer additional training to address workload challenges.
18	Tanaka et al (2024) (65) Japan	Improve health monitoring for the elderly through wearable technology.	The study finds that elderly participants are interested in wearable devices but face barriers like cost and complexity -It identifies four main themes: health issues, use of technology, and the advantages and disadvantages of wearable devices -The study concludes that these devices can benefit elderly individuals and require more research	-Simplify wearable designs and make them affordable -Ongoing training and technical support for both users and healthcare providers -Integrating wearables with existing healthcare systems and offering customizable features
19	Shagerdi et al (2022) (64) Iran	Enhance elderly care in emergency departments by implementing health information technologies to streamline workflows and improve collaboration	-The study finds no specific workflow for elderly care in EDs and suggests that tailored health IT solutions could improve care	-Create health information technology systems for elderly care in emergency departments and train staff on their use -More research is needed to assess the impact on patient outcomes and staff efficiency
20	Damery et al (2021) (51) United Kingdom	Improve the quality of care and patient safety in care homes through better practices, communication, and safety culture	-The SPACE program improved safety climate and reduced avoidable harms like falls and pressure ulcers -It also decreases emergency hospital attendances, though admissions increased -Upskilling staff enhances teamwork and safety practices, but high turnover may affect long-term sustainability.	-Address staff turnover and provide ongoing training to new staff -Reinforcing safety practices is essential for sustaining improvements -Expand the program to other care homes to offer further insights.
21	Read et al (2019) (63) Canada	Improve access to palliative care for elderly rural patients using web-based videoconferencing.	The study finds that web-based videoconferencing (WBVC) improves access to palliative care and is acceptable to patients, despite some technical issues -It saves time and travel, but raises concerns about data security -WBVC is a feasible alternative to in-person visits, but should complement them, not replace them.	-Improve provider training to reduce technical issues -Enhance technology to address quality and security concerns -Expand access to more rural areas to increase care reach
22	Kousha et al (2024) (53) Iran	Elderly care targets lonely community-dwelling older adults	The intervention reduces loneliness and improves quality of life through peer support and educational activities, and is a feasible and cost-effective way to combat loneliness in older adults	-Expand community involvement and digital literacy training to improve program reach -Increase follow-up support and add more activities to enhance effectiveness -Partnerships with local organizations can help sustain the program in the long term
23	Cahyanto et al (2023) (67) Indonesia	Improve the physical and mental well-being of residents through physical exercises and mental health activities	-The intervention improves participants' happiness, stamina, and sleep quality -Despite some challenges with balance exercises, the program is well-received with no injuries -It effectively enhances quality of life and can be sustained with accessible tools and consistency.	-Consider incorporating regular feedback to adapt the program and train more staff for better sustainability -Expand the program to include social interaction components, such as group activities or virtual meetings, which can further reduce isolation and improve overall well-being

CE, Continuing Education; CNCM, Community Nurse Case Manager; ED, Emergency Department; eHealth, Electronic Health; FC, Family Caregiver; GSFCH, Gold Standards Framework for Care Homes; HPDP, Health Promotion and Disease Prevention; HoSIP, Home Support Intervention Programme; HRQOL, Health-Related Quality of Life; IT, Information Technology; PCC, Person-Centred Care; PFP, Person First Please; PPC, Person-Patient Care; QI, Quality Improvement; QL, Qualitative; QT, Quantitative; RE-AIM, Reach, Effectiveness, Adoption, Implementation, and Maintenance; SAaH, Stay Active at Home; SHHC, Skilled Home Health Care; SHHCP, Skilled Home Health Care Provider; SPACE, Safer Provision and Caring Excellence; WBVC, Web-Based Videoconferencing.

#### Study context/sites

Eight studies - five quantitative (21, 46, 48, 53, 54); two qualitative (58, 62); and one mixed-methods (66) were single-site, while fifteen (nine quantitative; (47, 49–53, 57, 65, 67), four qualitative (56, 59, 61, 65); and two mixed-methods (51, 63) were multisite studies. Of the single-site studies, most were conducted in community health centers, local home care environments, or individual care homes, with one study using an embedded participatory design approach in a private home setting. The multisite studies spanned a range of contexts, including nursing homes across multiple regions, care home networks, primary care services, emergency departments, telehealth consultations across rural areas, and national healthcare improvement programs such as the Safer Provision and Caring Excellence (SPACE) and Gold Standards Framework for Care Homes (GSFCH). Several multisite studies also evaluated interventions across multiple hospital-to-home transitions and interprofessional settings, reflecting the complexity of care pathways for older adults (see Tables 1, 2).

#### Timing

The included studies span this timeframe and are presented by publication period; however, this reflects the distribution of the evidence rather than additional time-based inclusion criteria. The publication of these studies was divided into two time frames: 2017–2024 and 2004–2016. Nineteen studies- seven quantitative (46–48, 50, 53, 67), eleven qualitative (55–65), and one mixed-methods (51) - were published between 2017 and 2024. In contrast, six studies - four quantitative (21, 49, 54, 67), one qualitative (56) and one mixed-methods (66) - were published between 2004 and 2016. The recent body of literature reflects a growing interest in digital innovation, participatory design, and integrated care models for older adults (see Tables 1, 2).

#### Participants

##### Staff

Studies that involved health and social care professionals included home care nurses, nurse assistants, community nurse case managers (CNCMs), care aides, and home care staff (47, 51, 54–57, 59, 60, 63, 64). Interdisciplinary participants also included general practitioners (GPs), emergency department clinicians, geriatricians, and educators delivering simulation-based or web-based training (51, 52, 64) Additionally, some studies involved robotics engineers and technology developers, particularly those co-designing assistive technologies for use in the home (58) (see Tables 1, 2).

##### Patients

Studies that involved older adult participants included community-dwelling older adults, frail older adult individuals, and residents of care or nursing homes (48, 49, 53, 58, 62, 66, 67). Quantitative studies involved participants engaging with interventions such as structured discharge planning, weekly phone contact, or continuing education programs (21, 48, 52). Qualitative studies captured the perspectives of older people trialing wearable technologies or remote monitoring, as well as those involved in home-based robotic assistance (58, 60, 65). In several studies, family caregivers were also included, particularly where interventions focused on communication, safety, or shared care delivery in the home environment (57, 62, 66) (see Tables 1, 2).

### Types of interventions

All included studies were mapped against the operational definition of QI interventions described in the Methods section to ensure conceptual consistency. This review included 23 studies examining quality improvement (QI) interventions designed to support older adults across home, community, and residential settings. While some included studies explored user perceptions of technology, these were retained only where the technology was implemented as part of a broader quality improvement (QI) initiative or where findings directly informed implementation processes, consistent with established definitions of QI as structured efforts to improve care through applied change and evaluation (8). Interventions were categorized into five thematic areas based on their primary focus. Technology-based approaches (*n* = 9) were the most frequently reported, including remote monitoring systems, wearable devices, and assistive robotics (58, 61, 64, 65). Communication-focused interventions (*n* = 4) aimed to enhance coordination among care teams and with families (47, 57, 59). Education and training initiatives (*n* = 5) targeted workforce development through formal instruction, ongoing learning, and nurse-led care models (49, 52, 54). Co-design and planning interventions (*n* = 3), including structured discharge planning and participatory technology development, were reported in a few studies (21, 58, 66). Finally, peer support and social engagement strategies (*n* = 2) aimed at improving well-being and reducing loneliness (53, 67). This spread reflects a growing commitment to integrated and person-centered models of care across international contexts (see Tables 1, 2).

The five overarching categories represent the primary types of QI interventions identified in this review. The subsequent eight sub-themes are presented as complementary analytical dimensions that reflect how these interventions are implemented in practice, rather than as a strictly hierarchical extension of the five categories. This approach allows for a more nuanced understanding of both the nature of interventions and the strategies through which they are delivered.

#### Training: adopting or supporting the use of technology

This theme encompasses interventions aimed at upskilling staff or caregivers to adopt or support the use of technology. These programs frequently support both healthcare professionals and patients in engaging effectively with digital tools and electronic health (eHealth) systems. For instance, how home care nurses assess eHealth initiatives has been the topic of der Cingel (55). When incorporating technology into pure practice, the study emphasized the necessity of both technical proficiency and confidence, supporting training as a facilitator of person-centered digital care. The use of wearable health monitoring technology was also examined by author Tanaka et al., who emphasized the significance of usability and the role that training plays in patient and healthcare professional adoption (65).

Pigini et al. (66) also identified a connection between training and technology, as their user-centered design of robotic assistance technologies required awareness of user perceptions. Iterative feedback loops reinforced the need for training in the design process. All of these interventions are predicated on the idea that technology can improve the quality of care if properly supported by accessible, relevant training mechanisms.

#### Education: employing organized knowledge transmission

The primary aim of education-based interventions is to enhance healthcare delivery by employing organized knowledge transmission to healthcare providers or older individuals. These individuals frequently participate in ongoing professional development, illness prevention, or health promotion.

Using nurse-led health promotion strategies, Markle-Reid et al. (49) found that fragile older people’s health-related quality of life increased dramatically. Kajander-Unkuri et al. utilized web-based and simulation-based learning to enhance acute care assessments in home-care settings (52), while Pakkonen et al. (54) developed a continuing education (CE) program called “Person First Please” to assist nurses in developing their person-centered care (PCC) competency. Educational initiatives were also implemented in assisted living facilities, such as the Spirit program (67), which taught residents about physical and emotional wellness.

#### Communication: improving information flow

Effective communication is a crucial aspect of many interventions, as it improves information flow. These initiatives, whether among professional teams or between professionals and informal caregivers, are designed to reduce errors, increase care coordination, and enhance patient safety. For example, Petersen et al.’s (59) work on cross-sectoral communication between hospital and home care nurses highlighted the importance of understanding and aligning divergent practices and goals across sectors.

Meanwhile, Hoben et al. (47) have tested feedback mechanisms to improve internal team communication, specifically within care homes amongst staff members, including care aides and nurses. Macías-Colorado et al. (57) have all evaluated the exchange of safety information between community nurse case managers and family caregivers, identifying risks associated with unclear or inconsistent messaging. Although varying in context, these studies converge on the idea that poor communication compromises care, while structured, theory-informed interventions improve coordination and outcomes.

#### Technology/innovation implementation: assisting or monitoring older adults

Despite their differing settings, many studies agree that structured, theory-informed treatments enhance coordination and outcomes, providing reassurance and confidence in the effectiveness of these interventions. Yu et al. (61) and Shagerdi et al. (64) have all investigated long-term use and abandonment of technology by older adults, noting that usability, simplicity, and individual relevance were key to sustained adoption. ReadPaul et al. (63) used web-based videoconferencing for palliative consultations, which showed promise in rural settings despite concerns around security and technology reliability for the staff and patients alike. On the other hand, Keller et al. (60) and Vilstrup et al. (62) focused on home medical devices and iPads, respectively, revealing varying perceptions between healthcare providers and recipients. Across these interventions, the pattern is clear: technological success is mediated by user-centered design, simplicity, and appropriate training and support.

#### Person-centered care/client activation: ensuring patient autonomy, dignity, and tailored care

Person-centered care and client activation-based interventions have emphasized patient autonomy, dignity, and tailored care. This is often sought to change staff behavior or embed empathetic practices into care routines. For instance, Rooijackers et al. (50) designed training to shift staff behavior from “doing for” to “doing with” clients, promoting activation and self-. Dedhia et al. (21) have developed a structured discharge planning intervention aimed at ensuring transitions from hospital to home, reflecting patient-specific needs.

This philosophy was embedded in several studies, whose core goal was to realign care with the preferences, abilities, and values of the care recipient, fostering empowerment and inspiring shared decision-making. This approach instilled a sense of hope and inspiration by acknowledging the individuality and agency of older adults in their care.

#### Health and well-being promotion: enhancing the physical, psychological, or social well-being of older adults

Health and well-being promotion-based interventions aimed to enhance older adults’ physical, psychological, or social well-being. These included exercise programs, mental health activities, and strategies to reduce loneliness. For example, Light, Bishop (48) identified that the incorporation of structured weekly telephone contact significantly enhanced adherence to prescribed exercise regimens and contributed to measurable improvements in balance control among frail, community-dwelling older adults (48). Similarly, the study has found Davodi et al. (46) to have advanced the concept of active aging by implementing tailored interventions within health center settings, targeting both physical and social engagement of staff members. In parallel to these efforts, Kousha et al. (53) addressed psychosocial dimensions of older adult care by introducing a home-based support program that integrated peer engagement with educational components, thereby mitigating loneliness and enhancing quality of life for the patients.

Although these interventions vary in delivery mechanisms and specific focus, they are unified by a shared commitment to holistic care. Each approach foregrounds the interrelationships among physical health, emotional well-being, and social connectivity—key pillars for providing comprehensive, person-centered support for older adults. This comprehensive approach should reassure the audience that the care provided is thorough.

#### Systemic support: enhancing the organizational level delivery of care

These interventions represent strategic, system-level approaches to enhancing the organization and delivery of care, with a particular focus on improving safety, quality, and operational efficiency in care settings. A clear example of a system-level intervention is the SPACE program evaluated by Damery et al. (51), which combined staff upskilling with organizational support to improve safety culture in care homes and reduce avoidable incidents. In a related study, Hall et al. (56) investigated the application of the Gold Standards Framework in palliative care, drawing attention to both the practical challenges and the enabling factors that influence its successful integration into routine care.

Taken together, these studies emphasize the critical role of structural change in improving the delivery of care for older adults. They indicate that meaningful and sustained improvements in quality and safety are unlikely to result from isolated interventions. Instead, such improvements require broader organizational commitment, careful resource allocation, and a readiness to embrace changes to established practices.

#### Competence development/professional upskilling: strengthening the practical capabilities of healthcare staff

This theme of competence development and professional upskilling-based interventions focuses on strengthening the practical capabilities of healthcare staff in clinical settings. Although these are closely related to education, the emphasis here is on developing specific skills that can be directly applied in practice. These interventions may be assessed using tracking methods that change over time, such as pre- and post-intervention evaluations, to determine their effectiveness. For instance, Kajander-Unkuri et al. reported notable improvements in professional competence following a blended learning model that combined web-based and simulation-based training (52). Similarly, Damery et al. (51) found that the success of the SPACE program was closely tied to the upskilling of care home staff, which in turn contributed to improved safety practices. Moreover, the importance of staff competence in ensuring that technology-based interventions are adopted effectively (58, 65). The findings highlight that it is not enough for care professionals to be familiar with new technologies; they must also feel confident in their ability to use them effectively in day-to-day practice. If practitioners lack confidence, even the best-designed technologies can be overlooked or underutilized, limiting their impact in practice.

### Integrated synthesis

The integrated synthesis was structured to distinguish between (i) intervention types, (ii) measurable outcomes, and (iii) phenomena of interest related to implementation and user experience. This approach enabled a clearer mapping of how different QI interventions influence care processes, outcomes, and stakeholder experiences. These measurements are based on the main findings and claims of the included studies, which are briefly described hereinafter (see Tables 1, 2).

The synthesis emphasizes both measurable outcomes and phenomena of interest to provide a comprehensive understanding of the effectiveness and implementation of QI interventions. Focusing on outcomes and phenomena of interest reflects the mixed-methods design of this review, allowing the integration of quantitative evidence on intervention effectiveness with qualitative insights into experiences and implementation processes. In this review, ‘outcomes’ refer to measurable effects of QI interventions (e.g., improvements in safety, competence, or quality of life), while ‘phenomena of interest’ refer to experiential and contextual findings related to the implementation and perception of these interventions.

This synthesis follows a convergent integrated approach to mixed-methods systematic review, enabling the integration of quantitative and qualitative evidence into a unified analytical framework (43).

#### Outcomes of QI interventions (effectiveness)

Quantitative and mixed-method evidence demonstrated that QI interventions were associated with improvements in clinical outcomes, staff competence, patient safety, and quality of life, consistent with established evidence on the effectiveness of structured improvement approaches in healthcare (8).

##### Staff competence and self-efficacy

The study by Rooijackers et al. (50) on a reablement training program is a testament to the practical implications of staff self-efficacy, competence, and skill enhancement. Reablement, a term used to describe a person’s regaining of skills and abilities lost due to illness or injury, is the program’s focus. The program aimed to shift staff behavior from a passive “doing for” to an active “doing with” approach in supporting older adults. While the program did not lead to significant gains in staff self-efficacy or outcome expectations, it highlighted the practical relevance of reablement in supporting older adults more effectively. The findings suggest that training, in isolation, may be insufficient to bring about lasting behavioral change without the support of organizational backing, continued guidance, and mentorship (50). In contrast to self-efficacy, this report identifies several studies that address the enhancement of competence and clinical skills among staff across the sector. This finding was supported by Kajander-Unkuri et al. (52) who demonstrated that simulation-based learning improved nursing students’ clinical reasoning and preparedness for caring for older adults. Light et al. (48) found that structured fall prevention training increased staff knowledge and adherence to safety protocols in care home settings. Damery et al. (51) in their evaluation of the SPACE program, reported improvements in multidisciplinary team capability, including enhanced communication, care coordination, and increased confidence in managing complex cases. These findings underscore the value of well-designed training interventions in developing workforce competence and strengthening the delivery of safe, effective care.

##### Safety and risk reduction

Ensuring safety in the care of older adults relies not only on formal protocols but on the effective integration of people, systems, and environments. Damery et al. (51) demonstrated that the SPACE program strengthened safety culture in care homes, resulting in a reduction in preventable incidents, such as falls and pressure ulcers. Home-based care, as suggested by Macías-Colorado et al. (57) highlighted the increased risk of care failures when communication between nurse case managers and family caregivers is unclear or inconsistent, a challenge exacerbated by the involvement of multiple informal caregivers. Their findings highlighted a broader issue in community care: the need for clear and coordinated communication between formal and informal networks. Alongside Damery et al. (51) this study reinforces the view that safe, high-quality care depends not only on clinical protocols but also on relational continuity, mutual understanding, and organizational frameworks that actively support those delivering care in diverse settings. Complementing this, Davodi et al. (46) demonstrated how promoting active aging through digitally guided physical activity interventions could further enhance safety by reducing sedentary behavior, improving mobility, and preventing deterioration in functional health among older adults living independently.

##### Health-related quality of life

An integrated approach that encompasses not only clinical management but also the psychological and functional dimensions of well-being is essential to supporting older adults. Markle-Reid et al. (49) provided evidence that comprehensive, nurse-led interventions delivered in the home environment were particularly effective in enhancing health-related quality of life, especially among frail older individuals living in the community. This is especially true when the interventions are integrated into interprofessional frameworks. Social connection in later life is evident in Kousha et al. (53) who showed that peer-supported care models, in which older adults provided support and companionship to one another, played a significant role in promoting emotional well-being, primarily by reducing social isolation and enhancing feelings of autonomy among patients. Within institutional settings, Cahyanto et al. (67) reported that interventions integrating physical activity with mental health support yielded measurable improvements in energy levels, sleep quality, and psychological resilience. Davodi et al. (46) provided further support, demonstrating that engagement in technology-assisted aging programs promoted active participation and enhanced life satisfaction. Collectively, these findings underscore the importance of integrated, person-centered strategies in promoting overall well-being and quality of life among older populations.

##### Transitional care outcomes

Transitions from hospital to home represent a pivotal stage in the care continuum for older adults. Inadequate discharge planning constitutes a clinical risk with tangible consequences that extend beyond mere administrative oversight. Dedhia et al. (21) demonstrated that there is strong evidence that planned discharge protocols improve patient outcomes and reduce rehospitalization rates when they are applied with clinical foresight and interprofessional collaboration. This work highlights that effective discharge planning must be anticipatory, rather than reactive, and situated within a broader framework of transitional care. In parallel, Keller et al. (62) brought attention to the underexamined complexities of post-discharge life, highlighting the functional challenges older adults face when managing medical devices at home. Their results reveal a fundamental discrepancy between clinical discharge guidelines and the practical capacities of patients and caregivers, especially when environmental support or health literacy is limited.

##### Technology adoption

Evidence from quantitative and mixed-methods studies indicates that technology-based quality improvement (QI) interventions can improve care processes and outcomes for older adults. For example, technology-enabled interventions such as remote monitoring systems, assistive devices, and telehealth solutions have been associated with enhanced care coordination, increased service accessibility, and improved support for aging in place (46, 63). In addition, structured implementation of digital tools within care pathways has demonstrated potential to improve workflow efficiency and support clinical decision-making, particularly when combined with training and organizational support (51). However, the effectiveness of these interventions varies depending on the level of integration within care systems, with evidence suggesting that outcomes are optimized when technological solutions are embedded within broader, structured QI initiatives rather than implemented in isolation (8).

#### Peer support, social connection, and promoting active aging

Evidence from quantitative and mixed-methods studies demonstrates that QI interventions focusing on peer support and social engagement can significantly improve health and well-being outcomes among older adults. For example, social participation, particularly through peer-based initiatives, is increasingly considered essential to the agenda of ageing well. Kousha et al. (53) showed that programs rooted in mutual support within the home setting addressed social isolation and fostered a sense of purpose and agency among older adults, contributing positively to their overall well-being. Similarly, Cahyanto et al. (67) found that structured group activities combining physical and mental health components not only improved well-being but also encouraged sustained participation through a sense of community. Pigini et al. (66) further emphasized the value of involving older adults in the design of assistive technologies, noting that socially attuned and user-responsive systems can empower older individuals and promote independence. Together, these studies underscore the importance of social connection, not as a peripheral benefit, but as a fundamental component of successful aging interventions.

#### Experiences and implementation of QI interventions

Qualitative findings highlighted key phenomena influencing the implementation and sustainability of QI interventions, including communication, user engagement, contextual fit, and organizational readiness, which are recognized as critical determinants of successful quality improvement initiatives (9).

##### Communication and collaboration

The quality of communication between professionals across different settings significantly influences the effectiveness of collaborative care in later life. This was identified by Petersen et al. (59) who found that misunderstandings and role ambiguity between hospital and community nurses frequently disrupted transitional care, underscoring the need for clearer interprofessional communication frameworks and shared expectations. Hoben et al. (47) demonstrated that targeted feedback mechanisms could strengthen communication among frontline staff in nursing homes, particularly between care aides and registered nurses, thereby improving overall care quality. Macías-Colorado et al. (57) highlighted the risks posed by poor communication between community nurse case managers and family caregivers, especially in settings where responsibilities are informally shared. Similarly, the effective implementation of palliative care frameworks depended on open communication and mutual respect between care home staff and external providers (56). Together, these studies underscore that collaboration cannot occur in isolation - it requires ongoing, transparent communication embedded in everyday practice.

##### Co-designing with end users

Integrating end users into the design process has become a recognized imperative in the development of assistive technologies for older adults. Olatunji et al. (58) adopted a participatory methodology that embedded older individuals, alongside caregivers and engineers, within a collaborative design environment situated in the home. This collaborative approach not only enhanced the technology’s relevance to users’ daily lives but also strengthened their sense of agency and inclusion in the care process. The study underscored the value of design practices that lived experiences of older adults from the outset, ensuring that innovations were aligned with real-world needs rather than imposed from a purely technical perspective.

##### Person-centered care

Providing care that is responsive to the individual needs of older adults is not just a choice; it is necessary for safe and effective practice. Pakkonen et al. (54) found that professional training programs improved nurses’ ability to deliver care that reflected the personal values of those they supported. In contrast to this, Der Cingel et al. (55) noted that home care nurses were more likely to embrace digital health tools, such as remote monitoring systems or electronic health records, when these aligned closely with the specific clinical context of the patient, reinforcing the importance of personal relevance in both the human and technological aspects of care. Similarly, Dedhia et al. (21) emphasized the impact of tailored discharge planning, showing that when transitions from hospital to home were shaped around individual circumstances, outcomes were markedly improved. Olatunji et al. (58) extended this principle into the design of assistive technologies, demonstrating that involving older adults in shaping the tools intended to support them produced solutions that were more meaningful, usable, and empowering. Across these varied domains, the evidence consistently affirms that personalization is not merely a desirable feature of care but a critical foundation for improving quality, engagement, and dignity in later life. Similarly, it was shown that embedding older adults in the co-design of assistive technologies led to solutions that were better matched to their lived experiences and care priorities (58). Collectively, these studies underscore the significance of personalization as a driver of both engagement and efficacy in elder care. Taken together, these findings underscore the importance of personalization as a cornerstone of meaningful and effective care for older adults.

##### Barriers to implementation

Systemic barriers often constrain quality improvement in elder care. Hall et al. (56) identified staffing shortages, limited resources, and weak links with primary care as key obstacles to implementing palliative care in care homes. Similarly, der Cingel et al. (55) found that skepticism among home care nurses and a lack of alignment between eHealth tools and patient needs limited the uptake of digital interventions. Hoben et al. (47) further highlighted challenges in sustaining communication-focused initiatives in nursing homes, particularly when feedback mechanisms were not tailored to staff roles or organizational culture. Collectively, these studies suggest that implementation success depends not only on the design of the intervention but on structural readiness, practitioner engagement, and contextual fit within everyday care practices.

##### User experience

Qualitative evidence highlights that the success of technology-based QI interventions is strongly influenced by user experience and implementation context. Studies indicate that sustained engagement with digital tools depends less on technical functionality and more on perceived usability, accessibility, and personal relevance to older adults (61). Similarly, concerns related to affordability, complexity, and data security can limit the adoption of wearable technologies, despite their perceived benefits (65). The integration of technology into clinical practice is also shaped by how well these tools align with existing workflows, as poorly adapted systems may create additional burdens for healthcare professionals (64). Furthermore, while older adults may accept the use of digital tools such as tablets in care delivery, they continue to value face-to-face interaction and relational continuity, highlighting the importance of balancing technological innovation with person-centered care (60). Collectively, these findings demonstrate that successful implementation of QI interventions depends not only on the technology itself but also on contextual fit, user engagement, and ongoing support (9).

Mapping the findings across studies indicates that education and training interventions were consistently associated with improvements in staff competence and care quality, while communication-focused interventions enhanced coordination and patient safety. Technology-based interventions showed variable effectiveness, with outcomes highly dependent on usability, training, and contextual integration. System-level interventions demonstrated broader impacts on safety culture and organizational performance. This mapping provides a clearer understanding of how different QI strategies contribute to improvements in older care.

## Discussion

Although the success of QI interventions is dependent on clinician engagement and patient cooperation, QI initiatives in healthcare aim to enhance outcomes and address deficiencies (68). This review is designed as a mixed-methods systematic review that will incorporate evidence from and synthesize the results of single-method reviews (quantitative and qualitative), thereby producing reviews directly relevant to policymakers and practitioners. As this review covers a wide range of interventions and outcomes, along with the experiences and perspectives of both care providers and older individuals, the results are expected to reflect on and offer insights into some QI interventions and their impact, as well as common quality challenges in elder care. This review encompasses studies of individuals undergoing elder care, regardless of gender, diversity, ethnicity, socioeconomic status, or disability (13, 69).

QI interventions in older care, as evidenced by the success of a diverse array of approaches, are on a promising trajectory (33, 34). Each of these approaches, designed to enhance the quality of life and care experiences of older adults, reflects a shift toward more integrated and person-centered models of care (37, 70). The selection of 23 studies from 2,856 records identified through a systematic search is a testament to the growing interest and success in this field. These studies, which encompassed various designs and explored quality improvement interventions across eight thematic areas, including training, education, communication, technology, person-centered care, health promotion, systemic support, and professional upskilling, have shown significant improvements in operational efficiency, care coordination, patient engagement, and staff competence. Importantly, no adverse outcomes were reported by patients or staff, further reinforcing the success of these interventions.

Technology-based solutions, including wearable devices, assistive robotics, and videoconferencing platforms, have become particularly important among the various interventions. These tools not only enable remote monitoring but also promote greater user engagement and participation. However, the extant literature illustrates that the successful adoption and sustained utilization of these technologies were heavily dependent upon the provision of robust training and ongoing user support, both of which are essential to maximize their effectiveness and embed them meaningfully within care practices (55, 61, 65, 66). This is consistent with emerging evidence highlighting that the effectiveness of digital health interventions is strongly influenced by cultural relevance and contextual factors, particularly among diverse and underserved populations (71–74). Complementary to technological advances, educational interventions targeting both staff and older adults have demonstrated significant promise in enhancing clinical competence and promoting health literacy. Initiatives such as nurse-led health promotion (49), simulation-based learning (52), and person-centered care training programs (54) led to measurable improvements in care delivery and patient outcomes.

Promoting health and well-being also emerged as an important intervention domain, where programs targeting physical activity, mental health, and social connectedness demonstrated measurable improvements in quality of life (48, 53). Finally, systemic and competence development interventions, such as the SPACE program (51), highlight the essential role of organizational commitment and professional upskilling in fostering safe and sustainable care environments. The system-level interventions, as illustrated by the SPACE program (51), outline the essential role of organizational commitment in creating safety, cultivating a positive care culture, and strengthening workforce capability. Collectively, these approaches underscored the growing complexity and interrelated nature of strategies needed to provide person-centered, safe, and high-quality care for older adults.

Despite their variety, these interventions share several key similarities. Foremost is their commitment to improving care quality through enhancing clinical skills, streamlining communication, and empowering patients (47, 59, 62). Although technology plays a significant role in many interventions, thorough training and user assistance are necessary for its successful adoption (62). Person-centered care remains a key focus, with numerous programs designed to match support to older individuals’ values, skills, and preferences (55). Furthermore, communication emerges as a cross-cutting theme, with interventions frequently aimed at improving collaboration among professionals and between staff and patients or their families. This collaborative nature of the interventions makes everyone involved feel included and part of a greater effort.

However, these interventions also differ significantly in their design and focus. Some initiatives, such as systemic planning, aim to improve safety culture and operational efficiency at the organizational level. In contrast, person-centered and client activation interventions are more individually focused, aiming to foster autonomy and encourage self-management among older adults (50, 55). Some interventions are reactive, focusing on immediate needs like discharge planning, whereas others, notably health promotion initiatives, adopt a proactive approach to prevent decline (21, 49). This proactive nature of some interventions empowers the audience and makes them feel in control of the situation.

Together, these similarities and differences highlight the importance of a balanced and integrated approach (75). While each intervention has a distinct contribution, combining them can yield more holistic and sustainable improvements. For instance, integrating communication and person-centered principles into technological innovations may improve uptake and usability (58, 61). Systemic interventions are effective, especially when combined with staff development and patient engagement (51, 52).

The integrated synthesis has revealed that interventions for older adults may vary in design and overall implementation, ranging from technology-based tools to strategies for systematic organization. This ensured the commitment to improving care quality, promoting patient-centered care, and enhancing clinical effectiveness was unified and encompassed a common emphasis on communication, ongoing support, and training, particularly for the successful adoption of digital technologies (76, 77). Some interventions focus on changing systems and building staff capacity, while others aim to support older adults directly by promoting independence and self-care. This shows the importance of a joined-up approach that combines innovative use of technology with genuine, person-centered care, shaped by the wider system and the individual needs of those receiving support (78, 79).

The synthesis has revealed a shared commitment to enhancing the quality of care for adults. This is evidenced by various strategies, from total-system workforce development to individual empowerment through individual care. The review emphasized the value of combining digital tools with compassionate, tailored support to achieve meaningful, sustainable improvements in health outcomes.

### Strengths and limitations of the review

This review has notable strengths, particularly its inclusion of diverse study designs and international evidence, which together offer a comprehensive perspective on current interventions. While the review included a broad range of empirical study designs, it is acknowledged that certain approaches, such as realist evaluations, may not have been fully captured, which could limit insights into contextual mechanisms underlying QI interventions. The thematic synthesis, a method of synthesizing qualitative research that involves identifying, analyzing, and reporting patterns within the data, provides a useful framework for understanding how various strategies interact to influence outcomes. This review presents a comprehensive, critically engaged synthesis of quality improvement (QI) interventions relevant to the care of older adults across the sector, including home care environments and community health centers.

One of the study’s principal strengths lies in its adoption of the Joanna Briggs Institute (JBI) systematic review methodology (80). This methodology, which involves a systematic search, selection, and synthesis of existing knowledge, was used to map the evidence base for the review. Although resource constraints precluded full adherence to the protocol, the JBI framework provided a coherent and methodologically sound structure that underpinned the evidence mapping process for the individuals. This was supplemented by the Covidence systematic Review tool, a web-based software platform that streamlines systematic review production, to support data extraction and ensure consistency and thoroughness (45). The combination of the JBI systematic review methodology and the Covidence systematic Review tool facilitated a transparent, consistent, and replicable approach to identifying, selecting, and thematically analyzing the literature. This thorough process reinforces the review’s credibility and scholarly integrity for all readers, instilling confidence in the reliability of its findings.

The review’s international scope, drawing upon studies from diverse older care settings across 12 countries, underscores the global applicability of the report’s findings. This broad reach enhances the external validity, ensuring the findings are relevant to a broad audience. The thematic breadth, which encompasses key similarities across multiple interventions, further informs readers about the review’s wide impact and demonstrates a critical engagement with both the opportunities and potential risks associated with QI interventions in older care settings.

While the review has many strengths, it is essential to acknowledge certain limitations in a balanced and respectful manner. The study relied solely on secondary data from peer-reviewed English-language literature, which introduced potential language and publication bias. The restriction to English-language studies may have excluded relevant research published in other languages, such as Swedish, potentially limiting the comprehensiveness of the review (81). The most recent search was conducted in November 2024 and could not be updated due to resource constraints, resulting in the omission of relevant studies published thereafter. The report also acknowledges the absence of primary data collection or engagement with key stakeholders, specifically older adults, caregivers, and healthcare professionals, which constitutes a significant limitation. Such engagement may have yielded deeper insights into the contextual, ethical, and practical considerations of QI interventions in real-world care environments had it been more inclusive. This balanced approach to acknowledging limitations ensures that the review is honest and transparent, fostering trust with the audience.

### Future research directions

Future research should focus on evaluating and improving quality (QI) interventions, incorporating policy development and the systematic adaptation of older adult care. Specific emphasis should be placed on co-designed approaches involving stakeholders, with a focus on older adults, carers, and healthcare professionals. This will ensure that the interventions are contextually relevant to the individual as they will be ethically grounded and aligned with evolving care delivery models.

## Conclusion

The 23 studies in this review on approaches to older adult care examined the care they receive and their quality of life. The eight thematic areas of this study included: training, education, communication, technology, person-centered care, health promotion, systemic support, and professional upskilling. Technology-based interventions emerged as an important component of quality improvement strategies; however, their effectiveness was closely linked to factors such as training, user engagement, and contextual integration. Importantly, the review findings indicate that no single intervention type was sufficient in isolation, and that improvements in older care were achieved through a combination of approaches, including education and training, communication, person-centered care, health and well-being promotion, and system-level support. These findings align with the results of the integrated synthesis, which demonstrated that the effectiveness of QI interventions depends on their integration within broader care systems and their responsiveness to user and organizational contexts. Educational interventions for staff and older adults demonstrated promise in enhancing clinical competence and promoting health literacy through various initiatives. Areas that promote physical, mental, and social well-being have proven to improve quality of life. Systemic interventions exemplify the need for organization and ensuring suitable care environments. Because these areas differ, this illustrates the importance of utilizing them all for a more holistic approach. The JBI systematic review methodology should be adopted alongside the Covidence systematic review tool to enhance the review’s credibility. The absence of primary data from key stakeholders and older adults, as well as the exclusion of non-English reports, has resulted in limitations. Future research directions should prioritize QI interventions, with a particular focus on co-designed approaches involving stakeholders, to ensure that interventions are relevant and aligned with care delivery models.

## Data Availability

The original contributions presented in the study are included in the article/Supplementary material, further inquiries can be directed to the corresponding author.
