# Supplementary material for: Effectiveness and experiences of quality improvement interventions in older care: a mixed-methods systematic review
Source: Front Public Health. 2026 Apr 30;14:1813536. doi: 10.3389/fpubh.2026.1813536 (PMC13182273; doi:10.3389/fpubh.2026.1813536)
Supplement: Supplementary file 1 [file Table_1.docx]

| **Critical Appraisal Results using JBI Critical Appraisal Tools** | | | | | | | | | | | | | | |
| --- | --- | --- | --- | --- | --- | --- | --- | --- | --- | --- | --- | --- | --- | --- |
| **1-A Quantitative (RCTs)** | | | | | | | | | | | | | | |
|  | | | | | | | | | | | | | | |
| **Study** | **C1** | **C2** | **C3** | **C4** | **C5** | **C6** | **C7** | **C8** | **C9** | **C10** | **C11** | **C12** | **C13** | **% Yes**  **(Study Quality)** |
| Rooijackers et al (2022) | N | N | Y | N | N | U | N | N | Y | Y | N | Y | N | 31 (low) |
| Davodi (2023) | U | U | Y | N | N | U | N | Y | U | Y | Y | Y | Y | 46 (medium) |
| Hoben et al (2020) | Y | U | Y | N | N | U | Y | N | Y | Y | Y | Y | Y | 62 (medium) |
| Markle-Reid et al (2013) | Y | N | Y | N | N | Y | Y | Y | Y | Y | Y | Y | Y | 77 (high) |
| Light et al (2016) | Y | Y | Y | N | N | Y | Y | N | Y | Y | Y | Y | Y | 77 (high) |

**Table 1: Critical Appraisal Results for Included Studies**

C = criteria; N = no; N/A = not applicable; U = unclear; Y = yes.

C1–C13 represent the appraisal criteria based on the Joanna Briggs Institute (JBI) critical appraisal tools. The specific criteria vary according to study design (RCTs) and include domains such as methodological rigor, data collection, analysis, and validity.

| **1-B Quantitative (NRCTs)** | | | | | | | | | | | |
| --- | --- | --- | --- | --- | --- | --- | --- | --- | --- | --- | --- |
| **Study** | **C1** | **C2** | **C3** | **C4** | **C5** | **C6** | **C7** | **C8** | **C9** |  | **% Yes**  **(Study Quality)** |
| Pakkonen et al (2004) | Y | Y | N | Y | Y | N | Y | Y | Y |  | 78 (high) |
| Dedhia et al (2009) | Y | Y | Y | Y | Y | Y | Y | Y | Y |  | 100 (high) |
| Kajander-Unkuri et al (2021) | Y | N | Y | N | Y | N | Y | Y | Y |  | 67 (high) |
| Damery et al (2021) | Y | Y | Y | N | Y | N | Y | Y | Y |  | 78 (high) |
| Kousha et al (2024) | Y | N | N | Y | Y | Y | Y | Y | Y |  | 78 (high) |
| Pigini et al (2012) | U | Y | N | N | N | N | Y | U | Y |  | 33 (low) |

C = criteria; N = no; N/A = not applicable; U = unclear; Y = yes.

C1–C9 represent the appraisal criteria based on the Joanna Briggs Institute (JBI) critical appraisal tools. The specific criteria vary according to study design, NRCTs, and include domains such as methodological rigor, data collection, analysis, and validity.

| **1-C Qualitative Studies** | | | | | | | | | | | |
| --- | --- | --- | --- | --- | --- | --- | --- | --- | --- | --- | --- |
| **Study** | **C1** | **C2** | **C3** | **C4** | **C5** | **C6** | **C7** | **C8** | **C9** |  | **% Yes**  **(Study Quality)** |
| Yu et al (2023) | Y | Y | Y | Y | Y | U | U | Y | U |  | 67 (high) |
| Petersen (2019) | Y | Y | Y | Y | Y | N | N/A | Y | U |  | 67 (high) |
| Damery et al (2021) | Y | Y | Y | Y | Y | U | Y | Y | Y |  | 89 (high) |
| Olatunji et al (2024) | Y | Y | Y | Y | Y | Y | Y | Y | N/A |  | 89 (high) |
| Keller et al (2017) | Y | Y | Y | Y | Y | Y | U | Y | Y |  | 89 (high) |
| Pigini et al (2012) | Y | Y | Y | Y | Y | N/A | N/A | Y | Y |  | 78 (high) |
| Macías-Colorado et al (2021) | y | Y | Y | Y | Y | Y | Y | Y | Y |  | 100 (high) |
| DerCingel (2021) | y | y | y | Y | Y | U | Y | Y | Y |  | 89 (high) |
| Vilstrup et al (2017) | Y | Y | Y | Y | Y | Y | N | Y | Y |  | 89 (high) |
| Hall et al (2011) | Y | Y | Y | Y | Y | N | N | Y | Y |  | 78 (high) |
| Tanaka et al (2024) | Y | Y | Y | Y | Y | Y | U | Y | U |  | 78 (high) |
| Shagerdi et al (2022) | Y | Y | Y | Y | Y | N | N/A | Y | Y |  | 78 (high) |
| ReadPaul et al (2019) | Y | Y | Y | Y | Y | U | N | Y | Y |  | 78 (high) |
| Cahyanto et al (2023) | Y | Y | Y | Y | Y | N | N | Y | Y |  | 78 (high) |

C = criteria; N = no; N/A = not applicable; U = unclear; Y = yes.

C1–C9 represent the appraisal criteria based on the Joanna Briggs Institute (JBI) critical appraisal tools. The specific criteria vary according to study design (Qualitative) and include domains such as methodological rigor, data collection, analysis, and validity.
